# Double ipsilateral parathyroid adenomas, with one supernumerary and ectopic at the same time: a case report

**DOI:** 10.1186/s13256-021-02711-x

**Published:** 2021-04-13

**Authors:** Georgios Tzikos, Michael Polyzonis, Konstantinos Milias, Stefanos Milias, Ioannis Passos, Konstantinos Ioannidis, Nektaria-Dimitra Doutsini, Georgios Chatzoulis, Panagiotis Spyridopoulos

**Affiliations:** 1 1st Surgical Department, 424 General Military Hospital, Ring Road, 56429 EfkarpiaThessaloniki, Greece; 2 Pathology Department, 424 General Military Hospital, Thessaloniki, Greece; 3 Student of Medical, University of Sofia, Sofia, Bulgaria

**Keywords:** Primary hyperparathyroidism, Double adenoma, Ectopic, Supernumerary, Case report

## Abstract

**Background:**

Double adenomas (DA) represents a distinct clinical entity of primary hyperparathyroidism (PHPT). DA may follow various embryologic distribution patterns and could be supernumerary and/or ectopic.

**Case presentation:**

We describe the first case of PHPT which comes as a result of double ipsilateral adenoma, of which one was both ectopic and supernumerary. A 45 year-old Greek male patient with diagnosed PHPT due to a single lower right parathyroid adenoma was admitted to our department for surgical treatment. The preoperative tests (neck US, Sestamibi scan) were conclusive for single gland disease. The patient underwent focused parathyroidectomy. The frozen section revealed a parathyroid adenoma with a slight possibility for parathyroid carcinoma. Ten minutes after the excision, intact PTH (iPTH) dropped >50% related to preoperative values and was within normal range. Right hemithyroidectomy with additional ipsilateral central neck dissection was performed, because of the possibility for parathyroid carcinoma. The final pathology report showed that the first excised tissue proved to be a parathyroid adenoma, while a second subcapsular one and a normal right upper parathyroid gland were also found.

**Conclusions:**

Preoperative localization of DA using routine imaging tests and the utility of intraoperative parathyroid hormone assay are still unreliable in detecting multiple adenomas. Furthermore, a slight possibility of a second and simultaneously supernumerary and ectopic adenoma maybe present. Therefore, it would be advisable to establish the use of more advanced imaging tests (such as 4D-CT, 4D-MRI) or other diagnostic tools when DA are suspected.

## Background

Primary hyperparathyroidism (PHPT) is a common endocrine disorder. It is estimated that 100,000 new cases emerge every year in the United States of America. PHPT is characterized by excessive secretion of parathyroid hormone (PTH), causing calcium and phosphate abnormalities. The majority of PHPT cases are due to solitary benign adenoma (85-90%), while less frequently (15–20%) by either multiple adenomas or hyperplasia and rarely due to parathyroid carcinoma (1%) [1–3]. In recent years, there is a growing consensus that double adenomas (DA) represent a distinct clinical entity and should not be confused with an asynchronous type of four-gland hyperplasia. It is reported that the incidence of DA is up to 15% of all PHPT patients [4–7].

Ectopic or supernumerary parathyroid glands arise due to abnormal migration of parathyroid cells from the 3^rd^ and 4^th^ branchial pouches to their final position. Moreover, the incidence of one (or more) ectopic parathyroid adenoma(s) is between 6 and 16%, while the incidence of a supernumerary parathyroid adenoma is even lower, about 5% [8–10]. However, the incidence of an ectopic and supernumerary hyperfunctioning gland is only about 0.69% [11]. Milas *et al*. in their investigation of 127 patients with DAs found little prevalence of coexistence of DA with supernumerary or ectopic parathyroid gland though normal (4 and 3 out of 127 patients, respectively) [7]. Herein, we present the first case of PHPT due to ipsilateral DA, in which one was both ectopic and supernumerary.

## Case presentation

**Fig. 1:**
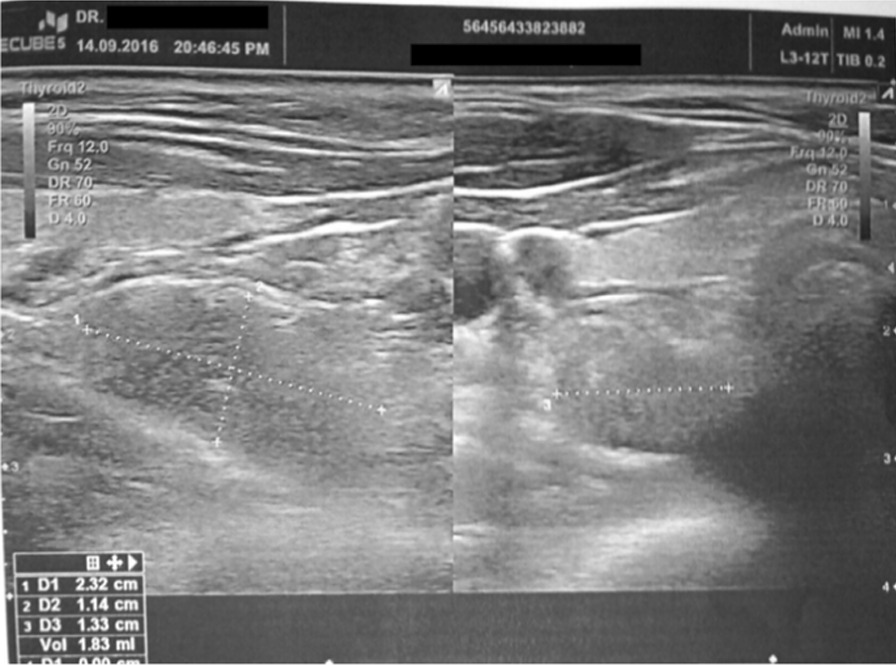
Thyroid ultrasound revealing an inferior right-sided nodule, indicative of parathyroid adenoma

**Fig. 2:**
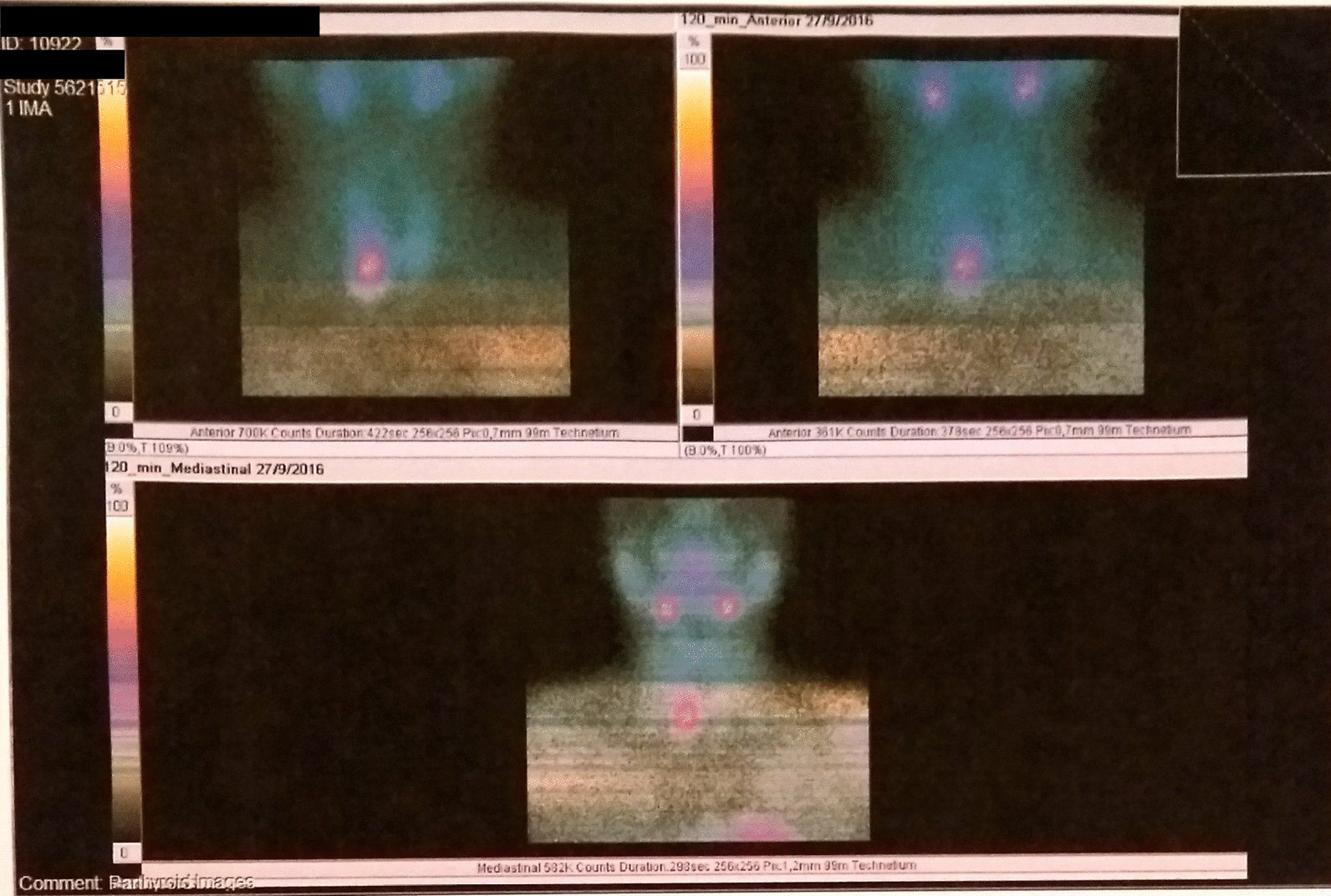
99mTc–Sestamibi scintigraphy revealing an inferior right-sided parathyroid adenoma

**Fig. 3:**
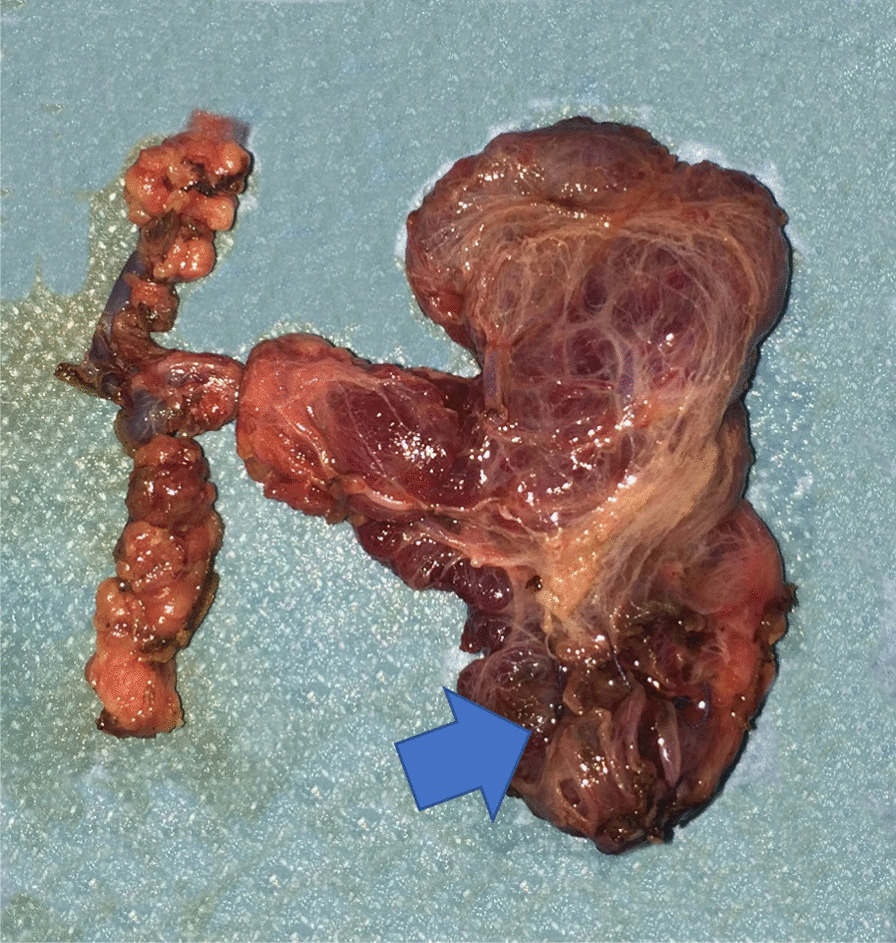
Right hemithyroidectomy specimen with additional resected nodes of right central compartment (the arrow shows the small bulging at the right lower thyroid pole)

**Fig. 4:**
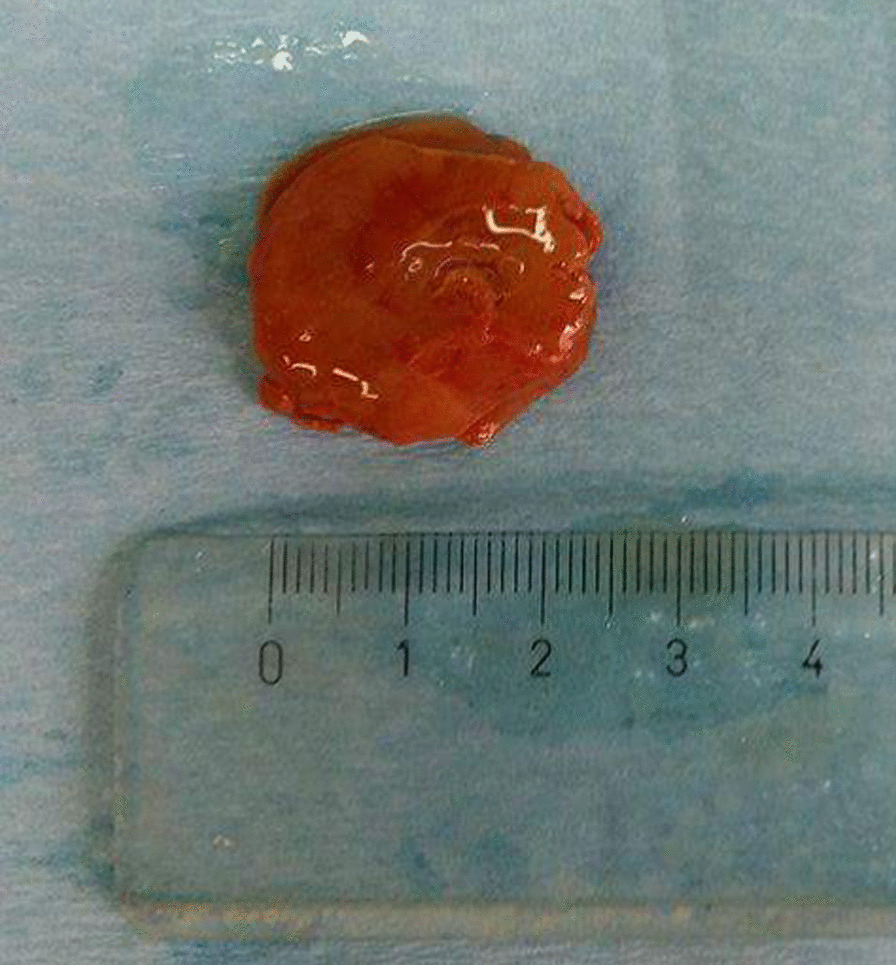
Excised ectopic parathyroid gland

**Fig. 5:**
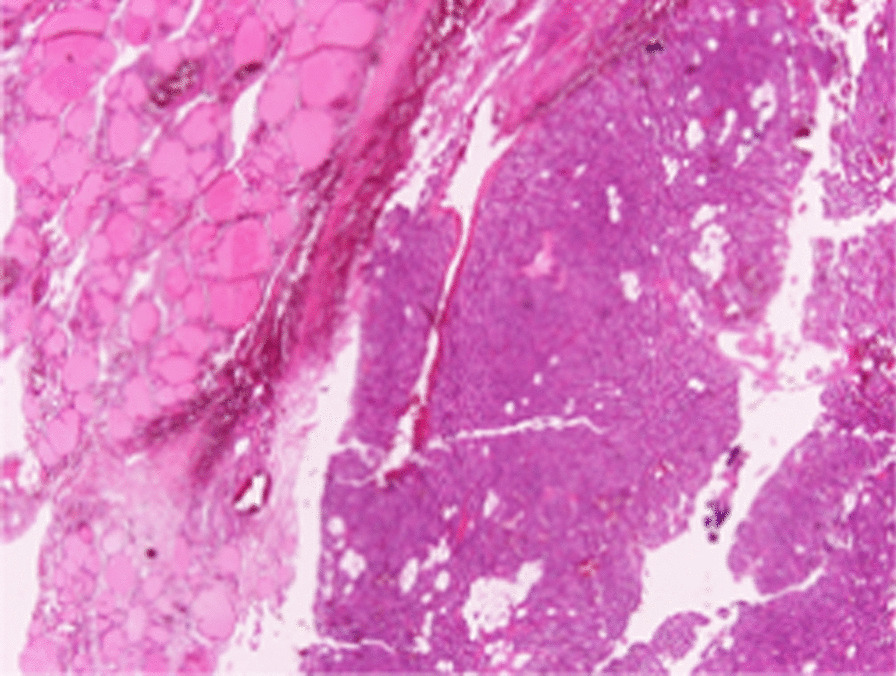
Pathology image of subcapsular parathyroid adenoma at the lower pole of the right thyroid lobe

A 45 year-old Greek male with PHPT admitted to our department for parathyroidectomy due to diagnosed PHPT caused by benign parathyroid adenoma. The patient was an Officer in Hellenic Air Force, married without children. On admission, he reported fatigue as the only symptom he experienced and the vital signs were normal (blood pressure: 135/65 mmHg, heart rate: 72 pulses/min, temperature: 36.8 °C). Our findings during the physical examination were the following: 1. head: eyes, nose, mouth, ears were normal, 2. neck: without any swelling area and trachea in the central position, 3. anterior torso: thorax and abdomen without abnormalities during observation, normal breath sounds, normal heart sounds and normal abdominal sounds, 4. posterior torso: without any spinal curvature or lung deformity, 5. pelvic and rectal examination: normal, 6. upper and lower extremities: without any abnormalities. Moreover, the results of our neurological examination were: 1. normal mental status, 2. cranial nerves evaluation: normal findings, 3. normal reflexes, 4. motor function and balance: normal findings, 5. sensory examination: normal findings, 6. coordination examination: normal findings. His medical history was free and there was not any family history for hypercalcemia or multiple endocrine neoplasia syndromes. The patient was not taking any medication before diagnosis. Besides, no allergies were reported and the patient did not smoke or consume alcohol. Laboratory tests revealed a serum total calcium of 2.8 mmol/l (normal range 2.05–2.55 mmol/l) with an elevated PTH of 204.70 pg/ml (normal range 15.00–68.30 pg/ml). Serum phosphate was 3.2 mg/dl (normal range: 2.5–4.5 mg/dl), 25 hydroxyvitamin-D level was 27.2 ng/ml (normal range: 20–50 ng/ml) and 24-h urine calcium level was 202.1 mg/24 h (normal range: 100–300 mg/24 h). Thyroid function tests were normal. The detailed laboratory results are reported in Table 1. Neck ultrasound revealed that the thyroid gland had normal dimensions and there was an inferior right-sided 23 x 11 x 13 mm extrathyroid nodule, indicative of parathyroid adenoma (Fig. 1). Furthermore, the patient underwent ^99m^Tc–Sestamibi scintigraphy (MIBI) and the result was in accordance with the neck ultrasound (Fig. 2). Under the diagnosis of PHPT and with a CaPTHUS score of 4 [Intact PTH level ≥ 2 times upper limit of normal PTH level, Sestamibi scan results positive for 1 enlarged parathyroid gland, Neck ultrasound results positive for 1 enlarged thyroid gland and concordant sestamibi and neck ultrasound study results (identifying 1 enlarged gland on the same side of the neck)] indicative for single gland disease, the patient was admitted for focused parathyroidectomy [12]. However, intraoperatively, we did not find any parathyroid gland at the lower pole of the right thyroid lobe. Therefore, we continued with a right neck exploration and only a normal parathyroid gland was recognized on the superior right pole of the thyroid. Thus, we decided to proceed on exploring the left side of the neck which revealed two normal parathyroid glands. Nevertheless, after repeated attempts to discover the pathologic right parathyroid gland, we noticed a small bulging at the right lower thyroid pole (Fig. 3: blue arrow) suspicious for an intrathyroidal parathyroid adenoma. Prior to the attempt to dissect this bulging, we finally found an enlarged nodule, morphologically similar to parathyroid adenoma. This was at the right side of the neck in connection with the carotid sheath, far away from the thyroid gland [Type C based on the nomenclature of parathyroid classification (Table 2) [13], which was excised and sent for frozen section (Fig. 4). Ten minutes after the excision of the suspected adenoma, a blood sample was taken and the intact PTH level dropped and was within normal range (49.30 pg/ml), indicating a reduction of more than 50% from the preoperative level. The frozen section report showed a “parathyroid neoplasm, with a low possibility of parathyroid carcinoma, due to the presence of fibrous bands”. Based on these findings, we decided to perform right hemithyroidectomy with additional ipsilateral central neck dissection (Fig. 3). In the immediate postoperative period and until discharge, paracetamol (1000 mg, four times per day), nonsteroidal anti-inflammatory drugs (lornoxicam 4 mg, twice per day) and omeprazole (20 mg, twice per day) were administered intravenously. The patient received and tolerated well liquid and solid food six hours after the procedure. In the first postoperative day, serum calcium, phosphate and PTH levels were 2.175 mmol/l, 1.04 mmol/l (normal range: 0.74–1.52 mmol/L) and 45.80 pg/ml respectively. Τhe patient discharged without any complications, in the second postoperative day. To our surprise, the final pathology report revealed that: (1) the first dissected “parathyroid neoplasm” was a parathyroid adenoma and not a carcinoma, with 2.7 cm maximum diameter, (2) there was one smaller subcapsular parathyroid adenoma, with 1.1 cm maximum diameter, located at the lower pole of the right thyroid lobe (Fig. 5) and (3) a normal parathyroid gland was found at the inferior border of the right central neck dissection specimen. Finally, eighteen months after surgery, patient’s serum calcium, serum phosphate and PTH levels were still within normal range (2.425 mmol/l, 1.06 mmol/l and 55.0 pg/ml respectively), confirming a successful, curative parathyroidectomy for PHPT. Furthermore, the patient did not report any symptom and physical and neurological examination did not reveal any pathological signs.

**Table 1:** Patient’s laboratory results on admission

**Test**	**Value**	**Normal values**
Complete Blood Count		
Hematocrit	45.4 %	42.0–54.0 %
Hemoglobin	15.0 gr/dl	13.0–18 gr/dl
Red Blood Cell Count	5.18 M/ml	4.5–5.5 M/ml
Mean Corpuscular Volume (MCV)	87.6 fl	78.0–98.0 fl
Mean Corpuscular Hemoglobin (MCH)	29.0 pg	27.0–31.0 pg
Mean Corpuscular Hemoglobin Concentration (MCHC)	33.0 gr/dl	32.0–36.0 gr/dl
Red Blood Cell Distribution Width (RDW-CV)	13.3 %	11.5–14.0 %
White Blood Cell Count	6.2 K/μL	4.0–11.0 K/μL
Neutrophils	62.0 %	40.0–70.0 %
Platelet Count	307 K/μL	142–450 K/μL
Biochemical tests		
Serum glucose	114 mg/dl	70–105 mg/dl
Serum urea	39.00 mg/dl	19.00–44.00 mg/dl
Serum creatinine	0.82 mg/dl	0.72–1.25 mg/dl
Aspartate transaminase (AST)	17.0 U/L	5.0–34.0 U/L
Alanine transaminase (ALT)	30.0 U/L	00.0–55.0 U/L
Gamma-glutamyl Transferase	20.0 U/L	12.0–64.0 U/L
Alkaline phosphatase (ALP)	70 U/L	40–150 U/L
Lactate dehydrogenase (LDH)	170 U/L	125–220 U/L
Serum potassium	4.5 mmol/L	3.4–5.1 mmol/L
Serum sodium	140.2 mmol/L	136.0–145.0 mmol/L
Triiodothyronine (T3)	1.2 ng/ml	0.6–1.6 ng/ml
Thyroxine (T4)	6.73 μg/dl	4.87–11.72 μg/dl
Thyroid Stimulating Hormone (TSH)	1.33 μIU/ml	0.35–4.94 μIU/ml
Serology		
Surface antigen of the hepatitis B virus (HBsAg)	0.19 S/CO	Negative < 1.00 S/CO
Hepatitis C Virus test	0.07 S/CO	Negative < 1.00 S/CO
HIV Ag/Ab	0.12 S/CO	Negative < 1.00 S/CO
Coagulation tests		
Activated Partial Thromboplastin Time (aPTT)	34.7 s	25.0–45.0 s
Prothrombin Time (PT)	11.31 s	12.00–14.00 s
International Normalized Ratio (I.N.R.)	0.95 1.00-1.50	

%: percent; gr/dl: grammar per decilitre; M/ml; millions per milliliter; fl: femtolitres; pg: picogrammars; K/μL: thousands per microlitre; U/L: Units per litre; mmol/L: millimoles per litre; ng/ml: nanograms per millilitre; μg/dL: micrograms per decilitre; μIU/ml: milli-international units per millilitre; S/CO: signal/cut-off; s: seconds

**Table 2:** Classifying parathyroid adenomas nomenclature [13]

Type	Location
A	Adherent to the posterior thyroid parenchyma. Lateral to the recurrent laryngeal nerve compressed within the capsule of the thyroid parenchyma.
B	A superior gland that has fallen posteriorly into the tracheoesophageal groove and is in the same cross-sectional plane as the superior portion of the thyroid parenchyma
C	Caudal to the thyroid parenchyma, in the tracheoesophageal groove. More inferior than a type B gland on lateral views. At the level of the inferior pole of the thyroid or inferior to the inferior pole of the thyroid (closer to the clavicle)
D	In the midregion of the posterior surface of the thyroid parenchyma, near the junction of the recurrent laryngeal nerve and the inferior thyroid artery or middle thyroidal vein
E	An inferior gland close to the inferior pole of the thyroid parenchyma, lying in the lateral plane with the thyroid parenchyma and anterior half of the trachea
F	An inferior gland that has descended (fallen) into the thyrothymic ligament or superior thymus
G	Truly intrathyroidal

## Discussion

In our study, we present the first described case of PHPT due to ipsilateral DA of which one was both ectopic and supernumerary and the other was subcapsular. We are demonstrating that DA represent a true discreet clinical entity which can contribute in difficulties or pitfalls concerning the management and the surgical plan of patients with PHPT. Although a lot of work has been done in order to identify the pathogenesis of adenomas and the difference between them and parathyroid hyperplasia, it is still controversial if adenomas arise only by clonal or also by polyclonal mechanism, even if most adenomas are of monoclonal proliferation [14]. However, it is clinically proven that DA represent a unique clinical setting and should be distinguished from hyperplasia. Medical literature report that there is a high long-term cure rate after two-adenoma resection in patients with PHPT, which is the best evidence of DA as a separate disease [4]. Moreover, a greater awareness of this entity among surgeons and a wider use of intraoperative parathyroid hormone assay (ioPTH) which helps in finding the adenoma and terminating the operation, have driven in a continuous rise of its initial prevalence, as high as 15% in recent studies [7].

Regarding the distribution of double parathyroid adenomas, it seems that they have a nonuniform pattern as Milas *et al*. observed in their study, although it was found that superior-bilateral DA were statistically more frequent (45%). Regarding the ipsilateral right DA, only 11 out of 127 (9%) patients had this configuration, although both adenomas were found to be orthotopic [7]. Similarly, Abboud *et al*. also found that DA can be identified in all possible configurations, with bilateral distribution being the most dominant [15]. Nevertheless, in our patient the distribution of DA was ipsilateral, even though one was ectopic**.** The inferior parathyroid glands arise from the 3rd branchial pouch and during their migration to their final orthotopic location, a number of factors, yet unknown, may affect their movement, contributing to the genesis of ectopic parathyroid tissue. In this case, our theory is that the parathyroid tissue of the 3rd pouch on the right, might have the tendency to evolve into a parathyroid adenoma. In addition, there has been an abnormal transportation of the parathyroid tissue of the 3rd pouch on the right, which resulted initially in its division, giving birth to the 5th parathyroid gland, and then in the migration of one of these two glands in an ectopic position. These two facts both contributed to the case of PHPT due to the DA that we have already described.

Concerning the size and weight of the DA, De Gregorio *et al*. found that two types of patterns have been identified: symmetric, in which both adenomas have almost the same size and weight and the asymmetric with one adenoma being heavier and larger than the other [4]. In our patient, the asymmetric pattern was recognized.

We believe that there are two main issues of great importance in our case, based on the global literature. The first is the inability of the preoperative exams to reveal the presence of both adenomas, while only one was detected. This difficulty in locating both adenomas, under ultrasound, sestamibi scan or a combination of them has been also documented in other studies and it is well known that DA are usually responsible for persistent or recurrent PHPT. A meta-analysis with 20,225 cases reported that although the sensitivity of MIBI and ultrasound was 88.44% and 78.55% respectively for single gland disease, although for the DA they were up to only 29.25% and 16.20% [16]. Furthermore, Haciyanli *et al*. reported that the accuracy in identifying double adenomas with ultrasound, sestamibi scan and the combination of them was only 40%, 30% and 60% respectively [17]. In addition, Abboud *et al*. in their study with 14 patients with DA also reported quite bad results of ultrasound and sestamibi scan detection ability and more precisely they found that the ultrasound of 11 patients failed to detect DA and so did the sestamibi scan in 10 of them [15]. Based on the aforementioned low sensitivity of MIBI and ultrasound to detect DA, we are able to explain why we failed to detect the second smaller adenoma. In our study, we collected imaging data indicative of single gland disease (both thyroid ultrasound and MIBI were positive for inferior right-sided adenoma). Another misleading point was the fact that CaPTHUS score was high (CaPTHUS score = 4), which shows high positive predictive value of around 96% for single gland disease. Besides, recent studies raise awareness of CaPTHUS score failure to predict single gland disease as a single preoperative modality and therefore ioPTH test was essential [18, 19]. As a result, we were quite convinced that PHPT was due to single gland disease. Therefore, we did not conduct any other, more advanced imaging tests, such as 4D MRI or F-fluorocholine PET/CT preoperatively, which are found to be more accurate in localizing ectopic or small parathyroid lesions and DA and so we proceeded to focused parathyroidectomy [20, 21]. In addition, we failed to find the orthotopic right inferior parathyroid adenoma during surgery, which was only revealed in the pathology report. The explanation for this failure was probably the fact that this adenoma (the small one) was under the thyroid capsule and in combination with its small size it was not visible during the examination of the right inferior pole of thyroid gland.

The second confusing point was that after the first adenoma excised, quick intraoperative PTH assay revealed a reduction in PTH levels more than 50% from the preoperative level, which was indicative of biochemical cure. A reduction of PTH level more than 35% is also predictive for single gland disease [22]. Thus, we believed that PHPT derived due to the excised single adenoma until we received the pathology report. The question that has to be answered is why the ioPTH levels dropped beyond the 50% cutoff point, although a second parathyroid adenoma was present. Kandil *et al*. in their study reported that intraoperative PTH levels failed to detect the presence of DA in 11 out of 20 PHPT patients (55%) [5]. In addition, in an American study including 21 patients with DA was found that in 12 patients (57%) ioPTH levels fell more than 50%, although only the first adenoma was resected and abnormal parathyroid tissue was still present [17]. Similar findings were reported also by Gordon *et al*., who found that the proportion of false positive results of quick intraoperative PTH assay as assessment tool for biochemical cure of PHPT during surgery were about 24% (4 out of 17 patients with DA) [23]. Moreover, in another study, ioPTH showed false positive decline in 9 out of 13 patients after the resection of the first adenoma when the first adenoma had greater weight than the second one [24]. The hypothesis behind this phenomenon is that one of the two parathyroid adenomas, usually the smaller, is suppressed due to the other hyperfunctioning adenoma and therefore, removing the second one results in false positive ioPTH level reduction, similar to single gland disease, as it probably happened in our case [25]. Another less likely theory introduced by Gauger *et al*. supports that the second adenoma is being devascularized during the process of its exploration or during the excision of the first adenoma and this is the reason why the PTH produced by that cannot enter the blood circulation and be detected [26]. In our patient we did not dissect the second adenoma at all as we were not aware of its presence. In addition, the prolongation of the operation was due to the small possibility of parathyroid carcinoma that the frozen section reported for the first adenoma.

We need to mention that during our study for this new case of PHPT and having searched the bibliography regarding DA thoroughly, we found that in 2013, Mazeh *et al*. suggested the Wisconsin Index (WIN) and the WIN nomogram. These were used in order to give an estimation of the likelihood that there is additional hyperfunctioning parathyroid gland during parathyroidectomy for PHPT, irrespectively if this is another adenoma or an overworking gland in the setting of hyperplasia. WIN is defined as the multiplication of preoperative serum calcium by preoperative PTH and the patients are divided into three categories: low (<800), medium (801–1600) and high (>1600). The combination of WIN and the weight of the parathyroid gland excised initially, may reveal the existence of an additional pathological gland. For glands weighting the same, the higher the WIN, the greater the possibility of another hyperfunctioning gland. Moreover, the weight of the parathyroid gland is reversely correlated with the chance of a second overworking one [27]. In our case, the WIN was 2292 which means that the possibility of a second hyperfunctioning gland is high. On the other hand, the weight of our heavier adenoma was 3000 mg, which decreases the possibility of a second adenoma. So, based on this article we could not estimate the possibility of a second adenoma clearly, because the chance of finding another adenoma correlates positively with the WIN but negatively with the weight based on the nomogram. In addition, a recent study demonstrates that there is a significant failure to cure PHPT in up to 13%, when only WIN is used to predict multi gland disease without ioPTH assay [28]. We are wondering whether it is worth reassessing the validity of the WIN index nomogram in future cohort studies in order to create a new more sophisticated system which will give us the possibility of a second pathologic parathyroid gland, after having calculated the WIN preoperatively, based on the weight of the first adenoma found intraoperatively.

## Conclusions

DA constitute a real unique clinical entity of PHPT and their distribution may vary widely, as in our patient, where the first adenoma was both ectopic and supernumerary and the second one was found under the thyroid capsule of the lower thyroid pole ipsilaterally. Their preoperative localization using the routine imagining tests is still rather disappointing as well as the adoption of ioPTH. In our patient we found a second parathyroid adenoma by chance and we showed for the first time that the possibility of ipsilateral DA of which the one is simultaneously ectopic and supernumerary does exist. For these reasons, we should consider the use of advanced imaging test such as 4D-MRI or F-fluorocholine PET/CT in highly suspicious for DA patients with PHPT. In order to assess these patients, a reevaluation of the suggested “Wisconsin index” nomogram or a similar modality, or even a combination of such a tool with ioPTH should be considered, in order to avoid losing DA. However, at the present time it may be almost impossible to identify preoperatively PHPT patients with DA, (with one adenoma not being localized in the usual imaging methods) who would be major candidate for more advanced imaging tests and the use of such imaging methods should be further investigated with cost-effectiveness cohort studies and randomized clinical trials.

## Data Availability

The data and materials/figures used in the current study are available from the corresponding author on reasonable request.

## Abbreviations

DA: Double Adenomas
PHPT: Primary Hyperparathyroidism
PTH: Parathyroid Hormone
iPTH: Intact Parathyroid Hormone
ioPTH: Intraoperative Parathyroid Hormone
MIBI: ^99M^Tc–Sestamibi scintigraphy
WIN: Wisconsin Index
4D-CT: Four-Dimensional Computed Tomography
4D-MRI: Four-Dimensional Magnetic Resonance Imaging

## References

[1] Kebebew E, Clark OH. Parathyroid adenoma, hyperplasia, and carcinoma: localization, technical details of primary neck exploration, and treatment of hypercalcemic crisis. Surg Oncol Clin N Am. 1998;7(4):721–48. 9735131

[2] Civelek AC, Ozalp E, Donovan P, Udelsman R. Prospective evaluation of delayed technetium-99m sestamibi SPECT scintigraphy for preoperative localization of primary hyperparathyroidism. Surgery. 2002;131(2):149–57. 10.1067/msy.2002.119817 11854692

[3] Kaplan EL, Yashiro T, Salti G. Primary hyperparathyroidism in the 1990s. Choice of surgical procedures for this disease. *Annals of surgery.* 1992;215(4):300-317. 10.1097/00000658-199204000-00002 PMC1242445 1558410

[4] De Gregorio L, Lubitz CC, Hodin RA, *et al*. The truth about double adenomas: incidence, localization, and intraoperative parathyroid hormone. J Am Coll Surg. 2016;222(6):1044–52. 10.1016/j.jamcollsurg.2015.12.048 27234627

[5] Kandil E, Alabbas HH, Bansal A, Islam T, Tufaro AP, Tufano RP. Intraoperative parathyroid hormone assay in patients with primary hyperparathyroidism and double adenoma. Arch Otolaryngol. 2009;135(12):1206–8. 10.1001/archoto.2009.192 20026817

[6] Alhefdhi A, Schneider DF, Sippel R, Chen H. Recurrent and persistence primary hyperparathyroidism occurs more frequently in patients with double adenomas. J Surg Res. 2014;190(1):198–202. 10.1016/j.jss.2014.02.024 24656398

[7] Milas M, Wagner K, Easley KA, Siperstein A, Weber CJ. Double adenomas revisited: nonuniform distribution favors enlarged superior parathyroids (fourth pouch disease). Surgery*.* 2003;134(6):995-1003 **(discussion 1003-1004)**. 10.1016/j.surg.2003.07.009 14668733

[8] Phitayakorn R, McHenry CR. Incidence and location of ectopic abnormal parathyroid glands. Am J Surg. 2006;191(3):418–23. 10.1016/j.amjsurg.2005.10.049 16490559

[9] Akerstrom G, Malmaeus J, Bergstrom R. Surgical anatomy of human parathyroid glands. Surgery. 1984;95(1):14–21. 6691181

[10] Wang C. The anatomic basis of parathyroid surgery. Ann Surg. 1976;183(3):271–5. 10.1097/00000658-197603000-00010 PMC1344236 1259483

[11] Henry JF, Defechereux T, Raffaelli M, Lubrano D, Iacobone M. Supernumerary ectopic hyperfunctioning parathyroid gland: a potential pitfall in surgery for sporadic primary hyperthyroidism. Ann Chir. 2000;125(3):247–52. 10.1016/s0003-3944(00)00247-9 10829504

[12] Kebebew E, Hwang J, Reiff E, Duh QY, Clark OH. Predictors of single-gland vs multigland parathyroid disease in primary hyperparathyroidism: a simple and accurate scoring model. *Arch Surg.* 2006;141(8):777-782 (discussion 782). 10.1001/archsurg.141.8.777 16924085

[13] Perrier ND, Edeiken B, Nunez R, *et al*. A novel nomenclature to classify parathyroid adenomas. World J Surg. 2009;33(3):412–6. 10.1007/s00268-008-9894-0 19148701

[14] Shi Y, Hogue J, Dixit D, Koh J, Olson JA Jr. Functional and genetic studies of isolated cells from parathyroid tumors reveal the complex pathogenesis of parathyroid neoplasia. Proc Natl Acad Sci USA. 2014;111(8):3092–7. 10.1073/pnas.1319742111 PMC3939880 24510902

[15] Abboud B, Sleilaty G, Helou E, *et al*. Existence and anatomic distribution of double parathyroid adenoma. Laryngoscope. 2005;115(6):1128–31. 10.1097/01.MLG.0000163745.57542.FE 15933536

[16] Ruda JM, Hollenbeak CS, Stack BC Jr. A systematic review of the diagnosis and treatment of primary hyperparathyroidism from 1995 to 2003. Otolaryngology. 2005;132(3):359–72. 10.1016/j.otohns.2004.10.005 15746845

[17] Haciyanli M, Lal G, Morita E, Duh Q-Y, Kebebew E, Clark OH. Accuracy of preoperative localization studies and intraoperative parathyroid hormone assay in patients with primary hyperparathyroidism and double adenoma. J Am Coll Surg. 2003;197(5):739–46. 10.1016/S1072-7515(03)00676-8 14585407

[18] Elfenbein DM, Weber S, Schneider DF, Sippel RS, Chen H. CaPTHUS scoring model in primary hyperparathyroidism: can it eliminate the need for ioPTH testing? Ann Surg Oncol. 2015;22(4):1191–5. 10.1245/s10434-014-4080-0 PMC4346409 25212837

[19] Mogollon-Gonzalez M, Notario-Fernandez P, Dominguez-Bastante M, *et al*. The CaPTHUS score as predictor of multiglandular primary hyperparathyroidism in a European population. Langenbeck’s Arch Surg. 2016;401(7):937–42. 10.1007/s00423-016-1426-z 27080995

[20] Memeh KO, Palacios JE, Khan R, Guerrero MA. Pre-operative localization of parathyroid adenoma: performance of 4d MRI parathyroid protocol. Endocrine Pract. 2019;25(4):361–5. 10.4158/EP-2018-0424 30720353

[21] Thanseer N, Bhadada SK, Sood A, *et al*. Comparative effectiveness of ultrasonography, 99mTc-Sestamibi, and 18F-Fluorocholine PET/CT in detecting parathyroid adenomas in patients with primary hyperparathyroidism. Clin Nucl Med. 2017;42(12):e491–7. 10.1097/RLU.0000000000001845 28902729

[22] Alhefdhi A, Ahmad K, Sippel R, Chen H, Schneider DF. Intraoperative parathyroid hormone levels at 5 min can identify multigland disease. Ann Surg Oncol. 2017;24(3):733–8. 10.1245/s10434-016-5617-1 PMC5292060 27743228

[23] Gordon LL, Snyder WH 3rd, Wians F Jr, Nwariaku F, Kim LT. The validity of quick intraoperative parathyroid hormone assay: an evaluation in seventy-two patients based on gross morphologic criteria. Surgery. 1999;126(6):1030–5. 10.1067/msy.2099.101833 10598184

[24] Sitges-Serra A, Diaz-Aguirregoitia FJ, de la Quintana A, *et al*. Weight difference between double parathyroid adenomas is the cause of false-positive IOPTH test after resection of the first lesion. World J Surg. 2010;34(6):1337–42. 10.1007/s00268-010-0413-8 20107797

[25] Zettinig G, Kurtaran A, Prager G, Kaserer K, Dudczak R, Niederle B. “Suppressed” double adenoma–a rare pitfall in minimally invasive parathyroidectomy. Horm Res. 2002;57(1–2):57–60. 10.1159/000057949 12006722

[26] Gauger PG, Agarwal G, England BG, *et al*. Intraoperative parathyroid hormone monitoring fails to detect double parathyroid adenomas: a 2-institution experience. Surgery. 2001;130(6):1005–10. 10.1067/msy.2001.118385 11742330

[27] Mazeh H, Chen H, Leverson G, Sippel RS. Creation of a “Wisconsin index” nomogram to predict the likelihood of additional hyperfunctioning parathyroid glands during parathyroidectomy. Ann Surg. 2013;257(1):138–41. 10.1097/SLA.0b013e31825ffbe1 22801087

[28] Edafe O, Collins EE, Ubhi CS, Balasubramanian SP. Current predictive models do not accurately differentiate between single and multi gland disease in primary hyperparathyroidism: a retrospective cohort study of two endocrine surgery units. Ann R Coll Surg Engl. 2018;100(2):140–5. 10.1308/rcsann.2017.0112 PMC5838681 29022783

